# Influence of Carbon Micro- and Nano-Fillers on the Viscoelastic Properties of Polyethylene Terephthalate

**DOI:** 10.3390/polym14122440

**Published:** 2022-06-16

**Authors:** Basheer A. Alshammari, Arthur N. Wilkinson, Bandar M. AlOtaibi, Mohammed F. Alotibi

**Affiliations:** 1 Material Science Research Institute, King Abdulaziz City for Science and Technology, P.O. Box 6086, Riyadh 11442, Saudi Arabia; mfalotaibi@kacst.edu.sa; 2 North West Composites Centre, Department of Materials, The University of Manchester, Manchester M13 9PL, UK; arthur.wilkinson@manchester.ac.uk; 3 The National Centre for Energy Storage Technologies, King Abdulaziz City for Science and Technology, P.O. Box 6086, Riyadh 11442, Saudi Arabia; bmalotaibi@kacst.edu.sa

**Keywords:** graphite nano-platelets, carbon nanotubes, DMTA, polymer composites

## Abstract

In this research study, three carbon fillers of varying dimensionality in the form of graphite (3D), graphite nano-platelets (2D), and multiwall carbon nanotubes (1D) were incorporated into a matrix of poly (ethylene terephthalate), forming carbon-reinforced polymer composites. Melt compounding was followed by compression moulding and then a quenching process for some of the samples to inhibit crystallization. The samples were analysed using dynamic mechanical thermal analysis (DMTA) and scanning electron microscopy (SEM), considering the dimensionality and loading of the carbon fillers. The dynamic mechanical analysis revealed a similar decline of storage moduli for all composites during the glassy to rubbery transition. However, storage moduli values at room temperature increased with higher loading of nano-fillers but only to a certain level; followed by a reduction attributed to the formation of agglomerates of nanotubes and/or rolled up of nano-platelets, as observed by SEM. Much greater reinforcement was observed for the carbon nanotubes compared to the graphite and or the graphite nano-platelets. The quenched PET samples showed significant changes in their dynamic mechanical properties due to both filler addition and to cold crystallization during the DMTA heating cycle. The magnitude of changes due to filler dimensionality was found to follow the order: 1D > 2D > 3D, this carbon filler with lower dimensionality have a more significant effect on the viscoelastic properties of polymer composite materials.

## 1. Introduction 

The use of carbon as a filler in polymer composites is widespread because of its multiple useful forms, including graphite, carbon black, graphene-based nanoparticles, and carbon nanotubes that are used to fabricate polymer matrix composites for a wide variety of applications. Conventionally, micron-size carbon fillers are used to improve the thermal, electrical, and mechanical properties of a polymer matrix. However, nano-size carbon fillers offer more significant improvement to polymer properties due to their much higher aspect ratios and surface areas compared with micro-fillers [1,2]. The nano-size carbon fillers have a large interfacial area and therefore a significant volume fraction of polymer develop interfacial characteristics with properties that vary from the polymer’s bulk properties even at a low loading of nano-fillers. The essential advantages of carbon nano-fillers over micro-fillers are the opportunities for improved multifunctional properties and decrease of the quantity of filler required to achieve desirable properties. Nevertheless, micro-fillers still are used to fabricate polymer composites due to their excellent mechanical properties, lower costs, and easier availability [3,4].

A common example of a micro-size carbon filler is graphite. Its 3D structure contains graphene sheets in a parallel arrangement having s_P_^2^ hybridized carbon that is bonded hexagonally. Graphite nano-platelets (GNP) are nano-scale carbon fillers prepared by modification of graphite, allowing certain atoms, molecules, or ions to be inserted between graphene sheets. In the graphene sheets, covalent bonds of very high strength are present between the atoms of carbon, whereas the parallel carbon sheets in graphite are connected through much weaker van der Waals forces [5]. Another common group of nano-scale carbon filler are the multiwall carbon nanotubes (MWCNT), multiple concentric cylindrical layers of graphene. Similar to graphite; the graphene layers in MWCNT are bonded by weak van der Waals forces of attractions which they arrange themselves into form of bundles and/or agglomerations. The bonding between carbon atoms within the tube walls is covalent bonding, which imparts great strength to weight ratio. The nanoscale conductive carbon fillers are classed as high aspect ratios (>1000) and great electrical, thermal and mechanical properties [6,7,8,9]. Therefore, they are ideal candidates for a wide range of applications including the development of nanocomposites materials. The main challenges to develop such materials is to enhance the dispersion and distribution of agglomerated MWCNT and/or folded/stacked GNP [10,11,12,13,14]. 

Several issues related to forming composites with carbon materials need to be addressed, including an interaction between polymer and carbon fillers, compatibility, dispersion quality, and non-uniform distribution. Therefore, pre-treatments are often required. To make them more reactive, functional groups can be attached as the reactivity is inhibited by their seamless nature [5,8,13].

Poly (ethylene terephthalate) (PET) is an aromatic, semi-crystalline engineering thermoplastic polyester, a common thermoplastic used in many applications due to its excellent properties such as transparency, wear and abrasion resistance, hardness, chemical resistance, recyclability, thermal and dimensional stability. It’s widely used for producing fibres and films for the packaging and textile industries [15,16,17,18,19]. However, most mentioned properties of PET are related to morphological and structural features such as orientation, degree of crystallinity (DoC) and the size and shape of crystallites. Numerous carbon fillers have been used to improve the properties and performance of PET and can play an important role in affecting mechanical, rheological, thermal, and electrical properties of the final composite [20,21,22,23,24,25,26,27,28,29,30].

Dynamic mechanical thermal analysis is an effective way to evaluate the viscoelastic behaviour of polymers and composites under dynamic conditions as a function of frequency or temperature. The mechanical response of polymers and their composites depends strongly on the testing time scale, due to their viscoelastic nature which also provides higher damping capacity compared to other engineering materials such as metals. The loss tangent damping parameter (tan δ) is very sensitive to molecular motions, transition relaxation processes and the morphology of composite materials. Therefore, to understand their behaviour at a molecular level, it is of great important to investigate the dynamic mechanical properties including storage modulus (E′) and tan δ. E′ indicates the elastic stiffness while tan δ specifies the amount of energy dissipated as heat during the deformation process [19,20,31,32,33]. 

Previous studies have carried out dynamic mechanical analysis on recycled PET films [17], metallized films PET [18], PET/carbon nanotube nanocomposite [21], PET/GNP nanocomposites [22,23], polyethylene/PET blends [34], interwoven hemp/PET hybrid composite [35], PET/hydroxyapatite composite [36] and, polypropylene/PET blend-montmorillonite nanocomposites [37]. These previous studies focused on the addition, separately and/or in combination of carbon fillers; mainly on comparing CNTs and laminar (graphene-like) nanomaterials. The properties of the resultant composites depended on the type, size, content, features and dimensions of these nanomaterials [38,39,40]. However, there is minimal research in the literature addressing a systematic evaluation of the effect of carbon filler dimensionality on the properties of thermoplastic composites. 

Therefore, the present study is designed to fabricate both micro-and nano-carbon composites using PET as the matrix with the incorporation of graphite (3D), GNP (2D), and MWCNT (1D) fillers to investigate the influence of filler geometry and loading on the dynamic mechanical properties and morphology of a PET matrix. The range of filler loadings were selected to extend from below to above their percolation threshold values, determined in our previous studies [24,25,26] to be ~14.7, 5.7, and 0.33 wt. % for the PET composites containing graphite, GNP, and MWCNT, respectively. 

## 2. Experimental

### 2.1. Materials

PET was received as pellets from Equi-polymers (LIGHTER C93) Germany; it had a glass transition temperature of 74 °C, a melting temperature of ~245 °C, and bulk density of 0.88 g/cm^3^. Synthetic graphite was purchased from Sigma-Aldrich (Gillingham, UK) as a powder with a particle size of <20 μm and a density of 2.2 g/cm^3^. The GNP (grade xGnP-M15), purchased from XG Sciences (Lansing, MI, USA), had a mean thickness and diameter of ~15 μm and 6–8 nm, respectively. The MWCNT used were NC7000 (Nanocyl), industrial grade MWCNT reported by the manufacturer to have an average diameter and length of 9.5 nm and 1.5 µm, respectively. Based on the average dimensions given for both MWCNT and GNP fillers in their suppliers’ data sheets, the calculated aspect ratios for these fillers are ~158 and ~1875, respectively.

### 2.2. Preparation of Composite Samples

Both the PET pellets and carbon fillers were dried at 120 °C in a vacuum oven overnight before processing. The PET/carbon composites’ preparation was carried out using a lab-scale twin-screw extruder (Thermo-Haake Minilab) in co-rotating mode. The mixing time, screw speed, and barrel temperature were maintained at 5 min, 45 rpm, and 280 °C, respectively. Extruded compounds were compression moulded at 280 °C for 10 min to form thin sheet (thickness = 1 mm), followed by quenching in ice-water bath to support the ductility and remove the brittle structure for easy processing. The compression-moulded samples, dried at 40 °C for 24 h, and stored for the dynamic mechanical analysis and morphological characterizations. The quenched PET sample was chosen for further analysis because it is ductile compared to slow-cooled sample, which is brittle, especially when fillers are added to it. 

### 2.3. Characterization of Composites 

Dynamic mechanical analysis was conducted using a DMA Q-800 (TA Instruments), in single cantilever mode. Specimens of dimensions 40 × 10 × 1 mm were heated from 23 to 220 °C at a heating rate of 3 °C/min under controlled cyclic strain (amplitude 10 μm, frequency 1 Hz). Storage modulus, E′, and loss tangent, tan δ, data were obtained as a function of temperature.

Scanning electron microscopy (SEM) was conducted using a Philips SEM XL30 at an accelerating voltage of 10–20 kV. The specimens were taken from the moulded films after being fractured using the tensile machine. The samples were fixed on 0.5-inch pin stubs (Agar Scientific, Stansted, UK) using carbon adhesive tape (Agar Scientific). The fracture surface morphologies of specimens were coated with a thin gold layer before SEM testing using an Edwards S150B sputter coater, to avoid charge formation on the specimen surface. The risk of charge formation on the surface was further reduced by using silver paint to form a conductive path between the specimens and the pin stub. 

For differential scanning calorimetry (DSC), a TA Instrument DSC Q100 was used to measure the DoC of the unfilled PET and its composites. Specimens (7–10 mg) were hermetically sealed in aluminium pans, and an empty pan was sealed and used as a reference. The specimens were scanned from room temperature to 280 °C in a nitrogen atmosphere using a 3-run heat-cool-heat programme, at heating and cooling rates of 10 °C/min. To erase their thermal history, specimens were kept at 280 °C for 5 min and then cooled down to room temperature. Three specimens from each material were measured and data obtained from all runs were used for analysis. 

## 3. Results and Discussions

### 3.1. Morphological Characterization of PET and Composites 

**Figure 1:**
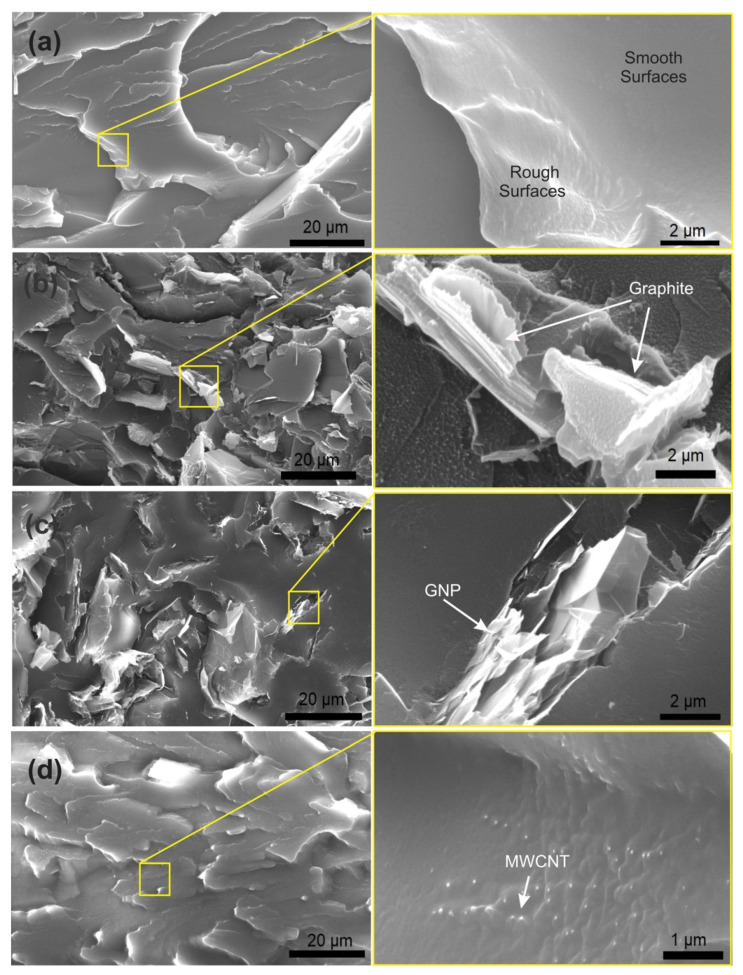
Fracture surface morphology with low(left) and high magnification (right) of (**a**) unfilled PET matrix, (**b**) PET/graphite microcomposites with 15 wt. % graphite, (**c**) PET/GNP nanocomposites with 10 wt. % GNP, and (**d**) PET/MWCNT nanocomposites with 0.1 wt. % MWCNT.

Figure 1 shows SEM images of tensile fracture surfaces of the unfilled PET (Figure 1a) and PET based micro- and nanocomposites, revealing different morphological features. 

In general, a rough fracture surface indicates more ductile behaviour with deformation of the matrix during tensile loading. On the other hand, a smooth surface indicates a more brittle fracture and less fracture toughness. The unfilled PET fracture surface appears smooth and rough, representing the brittle and ductile fracture, as shown in Figure 1a. It is clear from this Figure that the unfilled PET is free of air voids.

Images of the PET/graphite microcomposites at 15 and 2 wt. % loading of graphite is shown in Figure 1b and Figure S1. It is clear that as the graphite content was raised, the agglomeration level increased. De-bonding of the graphite from the PET matrix was observed at higher filler loading that possibly generated cracks resulting in composite failure. Corresponding fracture surfaces of PET/GNP nanocomposites are shown in Figure 1c and Figure S2 for 10 and 2 wt. % of M15 GNP, respectively. GNP agglomeration is absent at 2 wt. % of GNP, but some agglomeration, as well as rolling and folding up of sheets occurs as the GNP level is increased from 2 wt. % to 10 wt. %, as shown in Figure 1c. 

The GNP agglomerates formed at higher loading act as stress concentrators in the PET matrix and generate cracks. These images confirm that poor dispersion at 10 wt. % addition reduces the available interfacial area between the GNP and the PET matrix. The same results were reported by Akinci et al. [41] for polypropylene)/graphite microcomposites. In addition, Karevan et al. [42] observed a similar agglomeration effect at 12 wt. % GNP in a polyamide6 matrix. However, Larea et al. [27] reported that formation of micro voids and initiation of cracks from these voids was observed for PET/GNP nanocomposites with 5 and 10% wt. % but no sign of agglomeration was found in in their SEM images.

Figure 1d and Figure S3 show the fracture surface morphology of PET/ MWCNT nanocomposites at 0.1 and 1 wt. % MWCNT, respectively. At 0.1 wt. % MWCNT, good dispersion and distribution states of the nanotubes was observed whereas, in contrast, at 1 wt. % MWCNT both dispersion and distribution are inferior. CNTs exposed or pulled-out onto the surface are also evident in this figure, indicating relatively weak interfacial bonding with PET.

The weak interfacial bonding between the PET matrix and the carbon fillers is potentially attributed to the lack of active functional groups on the surface of the three fillers (MWCNTs, GNP, and Graphite). It is well known that the presence of function groups on the surface of fillers lead to a significant improvement in the interfacial bonding between the filler and the matrix in polymer composite systems. In order to confirm the presence of functional groups on the surface of these fillers, Fourier transform infrared spectroscopy (FTIR) was conducted as its spectra of used fillers can be seen in Figure S4. Comparing one to the other, all spectra show strong peaks in the ranges of 3000–3500 cm^−1^ which is a characteristic of hydroxyl (-OH) functional groups which is mainly due to moisture. In addition to the hydroxyl group, some minor peaks are present in all the three fillers. The peak in the ranges ~1700–1750 cm^−1^ suggests the existing of the carboxyl *group* (–COOH) [11,43,44,45,46]. It was previously reported that the attachment of -COOH groups onto the surface of carbon materials is of a great significance for strong bonding when compared to other functional groups [11]. In this study, it is clear that the presence of –COOH groups on the surfaces of the three fillers is trivial as shown in Figure S4. Therefore, the functional groups in this case did not play significant role to determine the mechanical properties of the investigated composite samples. 

### 3.2. Dynamic Mechanical Thermal Analysis (DMTA) Behaviour of the Unfilled PET Matrix

**Figure 2:**
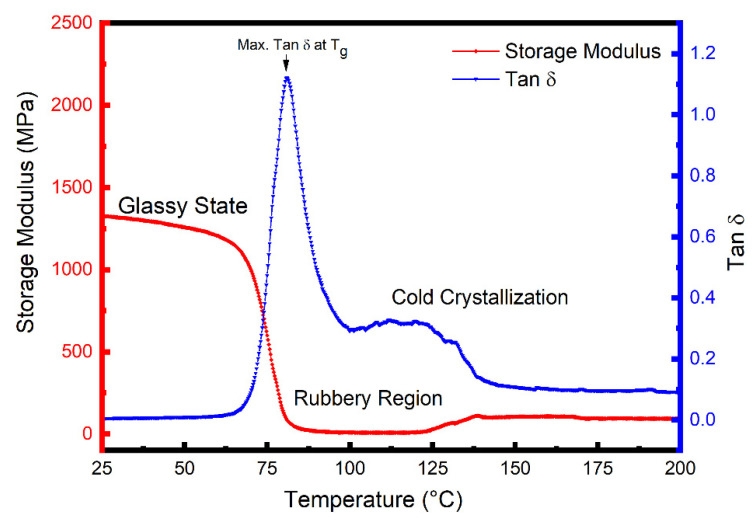
Dynamic mechanical thermal analysis (DMTA) curves of storage moulus, E′, and loss tangent, tan δ, as a function of temperature for the PET matrix.

Figure 2 shows E′ and tan δ (E′′/E′) data as a function of temperature for the quenched PET sample. The Figure shows that in this glassy state, the E′ values gradually decrease with temperature up to the T_g_, (≈80 °C); but as the temperature increases above T_g_ into the transition region the E′ decreases sharply from ~1330 MPa at 25 °C to reach a minimum value of 10 MPa at 100 °C, which is indicative of a glass-to-rubber transition of an essentially amorphous polymer. Above 100 °C, however, cold crystallization occurs during the DMTA heating scan, and the crystallites formed; as a result, increase the value of E′. The most unusual feature in the E′ curve of quenched PET samples is this influence of cold crystallization at temperatures above the T_g_, which was also observed for all the PET/carbon composites in this study. Similar behaviour has been reported previously for quenched unfilled PET [47] and PET nanocomposites [21,48]. Parvinzadeh et al. [48] and Bitenieks et al. [21] studied PET/clay and PET/CNTs nanocomposites, respectively, and attributed the observed increases in modulus above T_g_ to cold crystallization. 

Cold crystallization indicates that the quenched PET samples were not fully crystallized after processing and, therefore, crystallized during the first heating cycle in DSC, as indicated by the presence of a cold crystallization peak as shown in Figure S5.

For comparative purpose, the DoC of PET and its composite containing 2wt. % of each carbon filler was calculated from the first-heat data as these data reflect the processing thermal history of the materials. The DoC of PET and composite specimens were calculated using the following Equation [19,30]:

$$\mathrm{DoC}=\frac{\Delta H_{m}-\Delta H_{\mathrm{cc}}}{\left(1-w_{f}\right)\Delta H_{o}}\times 100$$

where $\Delta H_{m}$ is the melting enthalpy (J/g) measured in the heating tests,$\;\Delta H_{\mathrm{cc}}$ is the cold crystallization enthalpy (J/g), ∆H_o_ is the theoretical enthalpy of 100% crystalline PET ($\Delta H_{o}\;$= 140 J/g) and $w_{f}\;$is the weight fraction of carbon fillers. In order to confirm that the increase in E′ in the rubbery state is due to cold crystallization, a DSC test and a second DMTA run were conducted for the same (now crystallised) PET DMTA specimen, as shown in Figure S7. The insert in Figure S7 shows the DSC first heating run for this specimen, and it can be seen that the cold crystallization peak has disappeared. The DoC of the specimen after the first run of DMTA was found to be ~35% compared to ~11.8% obtained before the DMTA run. Moreover, no rise occurs in the E′ value during the second DMTA heating run for this crystallized sample and the E′ value at 100 °C (i.e., above T_g_ is about 530 MPa which is much higher than the value of ~10 MPa during the first run. These observations indicate that the quenched PET samples crystallized during the heating cycle of DMTA. 

### 3.3. DMTA Behaviour of PET/Graphite Microcomposites

**Figure 3:**
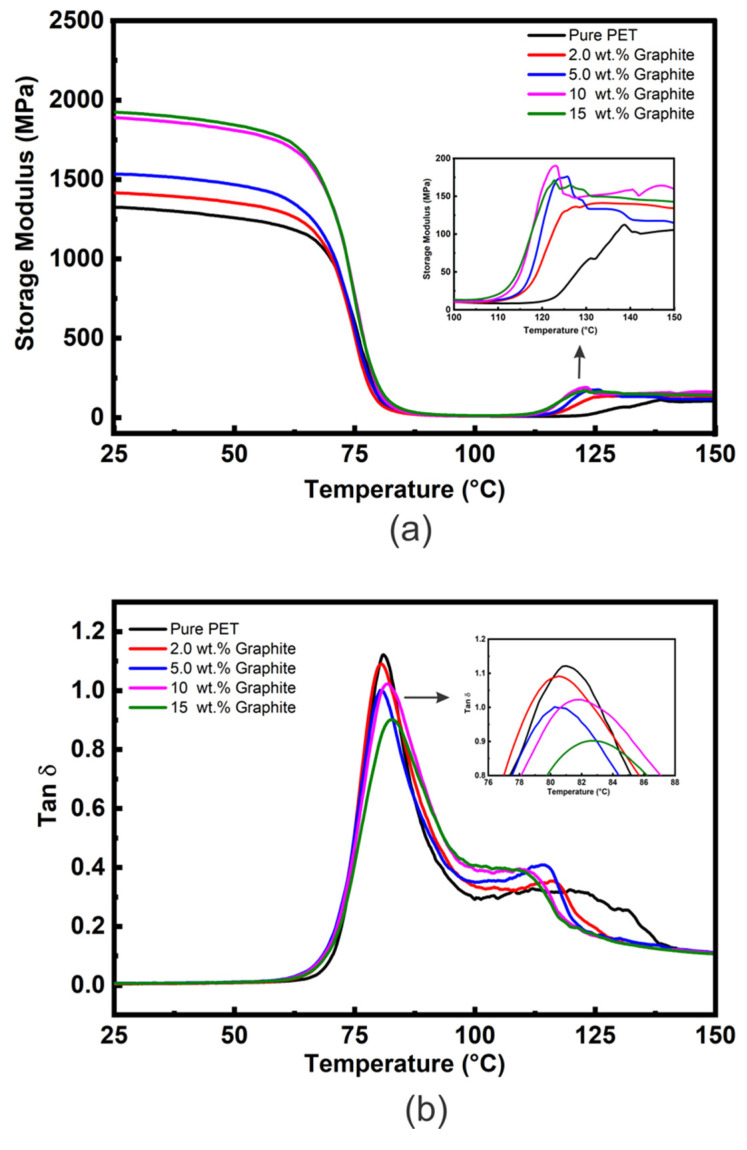
(**a**) DMTA E′ vs. temperature data and (**b**) DMTA tan δ vs. temperature data for the PET/graphite microcomposites.

The E′ vs. temperature curves for the PET matrix and microcomposites with varied loadings of graphite (2, 5, 10, and 15 wt. %) are shown in Figure 3a. Table 1 shows comparative E′ values at approximately room temperature (25 °C) and above T_g_ (100 °C). Figure 3a shows that the E′ curves for all the samples are essentially of the same shape; i.e., values of E′ (although different for each material all show relatively little reduction in the glassy region below T_g_ (~80 °C) and then decrease dramatically following the glass transition region reaching minimum values at ~100 °C. Additionally, upon rising the temperature above 100 °C a rise in the values of E′ for the microcomposites samples is observed in the rubbery region as shown in the insert Figure 3a. Again, this is due to cold crystallization during the DMTA heating cycle. It is also clear that the microcomposites’ cold crystallization begins at lower temperatures than for the unfilled PET, indicating that graphite nucleates the cold crystallization of the PET matrix.

Another observation from Figure 3a is that the E′ values of PET/graphite micro- composites below T_g_ increase with an increasing amount of graphite addition. The values of E′ at 25 °C for 2, 5, 10, and 15 wt. % graphite loadings increased by ~7, 16, 42, and 45%, respectively, compared to the unfilled PET matrix. Furthermore, type of polymer matrix affects the E′ values. For instance, diverse values of E′ at 25 °C have also been observed for PMMA/graphite microcomposites by Ramanthan et al. [45] and in PVDF/graphite micro composites by He et al. [46] despite they used same processing technique, i.e., solution method. Figure S8 shows E′ as a function of graphite content for polymer/graphite micro composites obtained from references [49,50] compared to the present experimental data. In general, it is clear that as graphite content increased, the E′ also increased. However, the improvement in the present study is much lower compared to the two previous studies. This could be due to different composite preparation methods (e.g., solvent vs. melt in the case of [49]) affecting the dispersion states of fillers in the matrices. The data in the literature is very scattered; for example, at 5 wt. % graphite, an ~18% increase of E′ at 30 °C was reported by Yasmin and co-workers [51] for an epoxy/graphite composite and a 25% increase for high density polyethylene (HDPE)/graphite composites by Zheng [52], both of which are much closer to the values observed in the present study. Whereas Zheng et al. [53] found that incorporation of 5 wt. % graphite had no significant effect on the E′ of a PMMA matrix. 

The tan δ vs. temperature plots for the PET/graphite microcomposites are shown in Figure 3b and data extracted from this Figure are summarized in Table 1. The T_g_ values indicate that no appreciable change occurs upon the addition of graphite, and similar results were observed by Yasmin et al. [51] for epoxy/graphite composites. However, these results contrast with Ramanathan et al. [49], who observed an increase in T_g_ of 30 °C for PMMA/graphite microcomposites containing 5 wt. % of graphite. Generally, an increase of T_g_ is attributed to segmental constraint due to interaction between the polymer matrix and the graphite particles. 

The damping response is a very dominant property of polymer composites and is directly related to tan δ values presenting the energy losses through segmental movements [54,55,56]. The tan δ values at T_g_ are inversely related to the volume of confined polymer within filler aggregates or interacting strongly with the filler’s surface, as such constraint hinders the mobility of chain segments. Figure 3b shows the tan δ peaks’ intensities decrease slightly and become broader as graphite content increases in the composites. The tan δ value of the microcomposites containing 15 wt. % graphite is about 0.88 which is ≈24% lower than that of unfilled PET (1.15); this is much greater than the reduction due to the replacement of polymer with ≈10% volume fraction of graphite and could be due to an increase in the DoC as well as to chain segment constraint in the interfacial region between the polymer and the filler [56]. Other peaks shown in the tan δ curves at higher temperatures ~110–140 °C reflect the cold crystallization. As graphite content increases, this peak’s maximum temperature decreases, although for the 10 and 15 wt. % loadings it becomes difficult to define their maximum temperature.

### 3.4. DMTA of PET/GNP Nanocomposites

**Figure 4:**
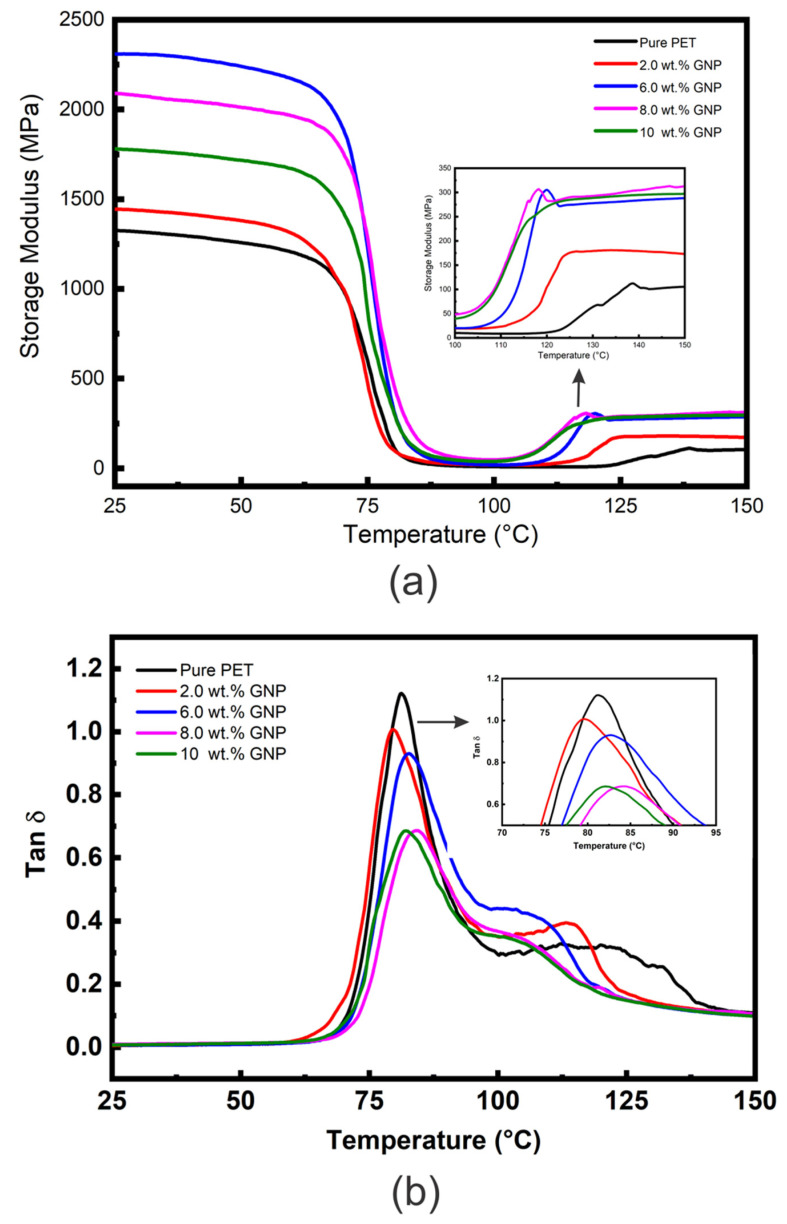
(**a**) DMTA E′ vs. temperature data and (**b**) DMTA tan δ vs. temperature data for the PET/GNP nanocomposites.

The E′ vs. temperature curves for the PET matrix and nanocomposites with different loadings of GNP (2, 6, 8, 10 wt. %) are presented in Figure 4a, and Table 2 shows comparative E′ values at approximately room temperature (25 °C) and above T_g_ (100 °C). Figure 4a shows curves of similar characteristic to those obtained for the PET/graphite composites (Figure 3a), the E′ curves for all the samples are essentially the same shape; i.e., values of E′ (although different for each material) all show relatively little reduction in the glassy region below T_g_ (~80 °C) and then decrease dramatically following the glass transition region reaching minimum values at ~100 °C. However, in contrast to the graphite microcomposites, the values of E′ are generally higher, indicating a greater degree of reinforcement and inducement of a greater DoC (See Figure S6).

Additionally, an increase the temperature above 100 °C, led to an increase in values of E′ for all nanocomposites samples is noticed, as shown in the insert of Figure 4a. Again this is due to cold crystallization of the amorphous regions developed during the quenching process. The cold crystallization of these GNP nanocomposites begins at lower temperatures than for the unfilled PET and for the graphite microcomposites. In addition, the increase in moduli following cold crystallization is much greater indicating that GNP nucleate cold crystallization of the PET matrix more efficiently than graphite, which may be due to their greater surface area.

Table 2 shows the maximum increase in E′ to be 72 % at 6 wt. % GNP. Incorporation of GNP beyond 6 wt. % into PET decreases the E′ values, but they remain higher than unfilled PET. The observed reduction could be due to agglomeration of GNP into PET matrix as has been observed in SEM image in Figure 1 for nanocomposites containing 10 wt. % of GNP which above their percolation threshold value [25], and therefore reduced dispersion and distribution at higher levels of GNP. GNP can also roll up during melt blending, as mentioned earlier, which reduces both their aspect ratio and interfacial area. The value of E′ at 10 wt. % GNP is very close to the value for graphite at the same wt. %, indicating that the effective modulus of the GNP (~1782 MPa) has reduced to that of graphite (~1893 MPa). Figure S9 shows comparative storage moduli as a function of GNP content of polymer/GNP nanocomposites obtained from the literature [13,28] compared with data from this study. All studies showed much closer trend for E′ values. However; higher E′ values have been reported by Ramanathan et al. [49]; which could be due to the a more efficient load transfer between PMMA and the GNP. Similar observations have been stated on the influence of GNP on the dynamic mechanical properties of polyester matrices, reporting enhancement of E′ (at ~25 °C) of 112 % and 66 % for PTT [57] and PBT [58] nanocomposites, respectively, at 7 wt. % GNP loading. Significant increases in E′ were reported by Aoyama et al. [22] who compared properties of PET nanocomposites containing GNP of two different layers (i.e., >3 layers and ≤3 layers). They achieved a 250% enhancement in E′ for PET upon addition only 2 wt. % of GNP (with ≤3 layers). Such improvement was ascribed to efficient load transfer from matrix to filler and high aspect ratio, resulting from a uniform filler distribution and good interfacial adhesion between the GNP and the PET matrix. Several studies [49,52,53] have compared the effect of graphite and GNP on E′ values and generally observed that the addition of GNP gave greater increases in E′ values than graphite, similar to the results in this study. This was attributed to the smaller size particles, higher aspect ratio, and larger surface area of the GNP compared to graphite. In addition to the mentioned factors that could affect the values of E′, type of polymer matrix play significant role in the E′ values of polymer/GNP nanocomposites as reported previously [5]. 

Figure 4b shows tan δ vs. temperature data for the PET/GNP nanocomposites, and Table 2 summarises T_g_ and tan δ values obtained from this figure. It is clear that the intensity of the tan δ peaks declines for the nanocomposites compared to unfilled PET. For example, the tan δ values reduced 40% (from ~1.15 to 0.69) for unfilled PET to nanocomposites containing 8 wt. % GNP, significantly greater than the reduction due to the replacement of polymer with ≈5.2% volume fraction of GNP. The reduction in tan δ values could be attributed to the increase in the DoC from 11.8% (unfilled PET) to 22% for the PET nanocomposites and to constraint of PET segmental mobility at the matrix-GNP interface. It has been reported that the area under tan δ peaks, usually decreases with increasing GNP loading [59], which is attributed to the 2D structure of the graphene sheets which hinder the segmental transition from the glassy to the rubbery state. The reductions in tan δ values for the GNP are much greater than those observed for graphite; for example, the reduction at 10 wt. % is −0.46 (−40%) for GNP compared to −0.13 (−11%) for graphite. This reflects the difference in specific surface area between the two carbon fillers.

The DMTA results of the current study validate previous studies [59] regarding enhancement of E′ and reduction of tan δ values upon incorporating GNP within polymer matrices. However, the T_g_ values of PET/GNP nanocomposites show no appreciable change (Table 2). Similar observations of T_g_ and tan δ have been reported for different matrices, such as PET/GNP [55], PTT/GNP [57], and PBT/GNP [58] nanocomposites. They attributed this behaviour to higher filler surface area and the possibility of functional groups that can promote the attraction of PET chain segments onto the GNP surface, thereby restricting their mobility.

### 3.5. DMTA of PET/MWCNT Nanocomposites 

**Figure 5:**
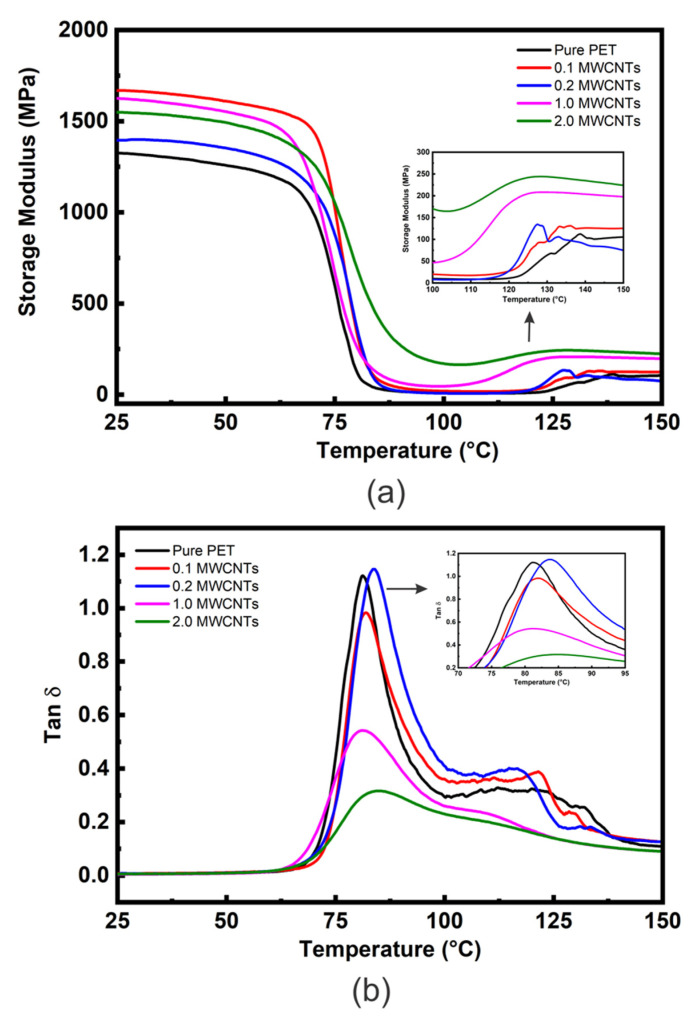
(**a**) DMTA E′ vs. temperature data and (**b**) DMTA tan δ vs. temperature data for the PET/MWCNT nanocomposites.

The E′ vs. temperature curves of the PET matrix and nanocomposites with different loadings of MWCNT (0.1, 0.2, 1 and 2 wt. %) are shown in Figure 5a and Table 3 shows comparative E′ data at approximately room temperature (25 °C) and above T_g_ (100 °C). Figure 5a shows E′ curves similar in shape to those in Figure 3 and Figure 4; i.e., all values of E′ (although different for each material) show relatively little reduction in the glassy region below T_g_ (~80 °C) and then decrease dramatically following the glass transition region reaching minimum values at ~100 °C. Similar behaviour was also reported by Bitenieks et al. [21], who studied the dynamic mechanical properties of PET/MWCNT nanocomposites. The study reported that cold crystallization increased E′ of the composites above T_g_. However, E′ increased by 8, 18 and 300 times upon addition of 1 wt. %, 2 wt. %, and 5 wt. % of CNTs, respectively. 

However, in contrast to the GNP nanocomposites (Figure 4a), the values of E′ are generally much higher at additions of at 2 wt. %, indicating a greater degree of reinforcement in addition to the inducement of a greater DoC (from 19.3 to 22.5 wt. % for the 2 wt. % nanocomposites—see Table S1). Above 100 °C an increase in E′ values due to cold crystallization is observed for all the nanocomposites, as shown in the insert Figure 5a. The MWCNT nanocomposites’ cold crystallization begins at lower temperatures than for the unfilled PET, indicating nucleation by the MWCNT. The nucleation effect of 1 wt. % nanotubes appears similar to that of 2 wt. % of GNP. However, the nanocomposites containing 2 wt. % nanotubes show a very high modulus at 100 °C of 169 ± 43 MPa (compared to approximately 30 MPa for 2 wt. % GNP).

It is clear from Figure 5a and Table 3 that all nanocomposites possess higher E′ values than unfilled PET. For example, a MWCNT loading of only 0.1 wt. % increases E′ by ~26%. This could be attributed to the high modulus of CNTs (~1 TPa) and their good dispersion and uniform distribution into the loading matrix. 

Increased addition to 0.2 wt. % of MWCNT, decreased the value of E′ from 1668 MPa to 1397 MPa (−16%), however, most of this reduction was recovered upon increasing the loading to 1 and 2 wt. %. The general reduction in reinforcement above 0.1 wt. % can be attributed to entanglement/agglomeration of MWCNT and their non-uniform distribution into the PET matrix [8], as observed with SEM, which reduces their aspect ratio and the surface area of their interface with the matrix. The enhancement of E′ is more distinct in the rubbery state, especially at 2 wt. % addition, as shown in Figure 5a and Table 3. This may be due to forming a rigid percolating network within the PET matrix as the MWCNT content increases above the percolation limit for this system of 0.33 wt. % [24,25,26] and CNT-CNT interactions become dominant. Figure S10 shows some E′ literature values for MWCNT-based nanocomposites compared to those of the present study. 

Bitenieks et al. [21] reported that E′ of PET/MWCNT increased by ~7% upon the incorporation of 2 wt. % MWCNT compared to unfilled PET, with the increase reaching ~20.8% at 5 wt. % MWCNT (Figure S10). The CNTs were reported to be dispersed homogenously and well distributed within the PET matrix; with no sign of agglomeration. Amoroso et al. [60] reported that the addition of 0.3 wt. % MWCNT into HDPE produced an increase in E′ by 16% with no agglomerations observed. For addition above 0.3 wt. % of MWCNT only very slight increases were observed. This behaviour was attributed to poor interfacial interaction between the HDPE matrix and CNTs filler resulting in CNT agglomeration and entanglement. Logakis et al. [61] reported a similar trend to the current study (Figure S10). Both studies report an abrupt improvement in E′ values at low levels of MWCNT addition, followed by a decline at higher loadings. The reduction is attributed to CNT entanglement/agglomeration. A comparison between MWCNT- and carbon black-PP composites in terms of dynamic mechanical properties was reported by Manchado et al. [62], who observed a dramatic improvement in E′ for both fillers. However, they also noted a significant decrease in the modulus when CNT loading exceeded 0.75 wt. %. On the other hand, when carbon black was used, the modulus gradually increased, and no reduction occurred despite some agglomerates forming in the PP matrix. This was attributed to the diverse interfacial areas and shapes of the fillers used. 

Variations in E′ values have been reported for several polymer/CNT systems mainly dependent on the dispersion state, interfacial adhesion, preparation methods, CNT surface modifications and type of CNT and their content as well as type of polymer matrix [8,10,21,30,61,63]. For example, Bitenieks et al. [21] and Logakis et al. [61] used similar processing techniques and CNTs but they reported different improvement in E′ values, i.e., 7% and 77% for PET and PP matrix composite, respectively. This could be due to in fact that the type of polymer matrices. Moreover, the reactivity of the filler with the polymeric matrix plays significantly on the E′ values. Therefore, it further suggests that the evaluation of chemical characterization of the filler surfaces maybe is required to know of the percentage of improvement is attributed to such reactivity. 

**Figure 6:**
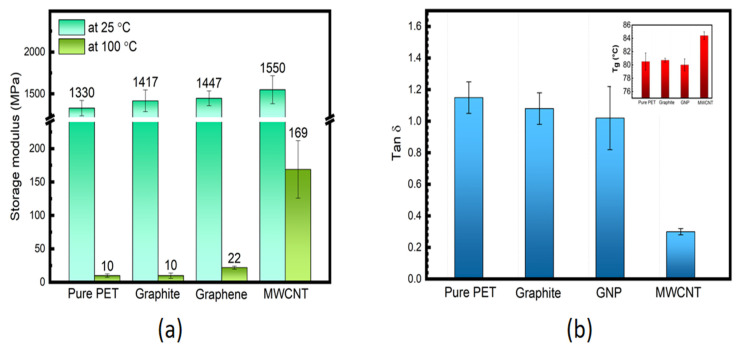
(**a**) Comparison of E′ at 25 and 100 °C and (**b**) tan δ at T_g_ (T_g_ shown in the inset of unfilled PET and composites containing 2 wt. % of graphite, GNP and MWCNT; respectively).

The tan δ vs. temperature data for PET/ MWCNT nanocomposites are shown in Figure 5b. The T_g_ and tan δ values at T_g_ extracted from this Figure are reported in Table 3. Figure 5b shows the tan δ peak intensities to decrease slightly and become broader as MWCNT content is increased in the nanocomposites except at 0.2 wt. % (tan δ ≈ 1.16), which has already been identified as a possible irregularity presenting similar values for unfilled PET (tan δ ≈ 1.15). In contrast, the tan δ value of the nanocomposite with 0.1 wt. % of MWCNT is about 0.98, which is ≈15% lower than for unfilled PET. The materials with higher loadings showed a sharp reduction in tan δ values, i.e., down to 0.55 and 0.31 and 2 wt. % MWCNT, respectively, as shown in Table 3. For comparison, Figure 6a shows values of E′ at 25 and 100 °C for composites containing the same wt. % loading of carbon fillers. It’s clear that the value of E′ at 2 wt. % CNT_S_ is very close to the value for GNP and graphite at the same wt. %, indicating that the effective modulus of the CNT has reduced to that of graphite. In addition, the tan δ value of 0.3 at 2 wt. % for the CNT nanocomposite is significantly lower than for the equivalent GNP nanocomposite (1.02) (see Figure 6b). This behaviour is often ascribed to an increase in the DoC and/or to more significant chain segment constraint in the interfacial region between the MWCNT and the PET matrix. As the DoC in the 2 wt. % MWCNT nanocomposite is not significantly different to that of the 2 wt. % GNP composite (≈3.5% greater) crystallinity and may be ruled out (Figure S5). Given that the degree of reinforcement provided by both the MWCNT and GNP at 2 wt. % addition appears similar (their values of E′ are within 4%) differences in interfacial interactions seem unlikely, which are very dominant. A plausible cause of the significant decrease in tan δ at 2 wt. % MWCNT is the formation of a percolated CNT network, which would impose a significant constraint on molecular movement. It has been reported that the degree of reinforcement is determined by the structure of the filler in the polymer matrix [64,65].

Other peaks shown in the tan δ curves at higher temperatures ~110–140 °C reflect the cold crystallization process. As MWCNT content increases, this peak’s maximum temperature decreases, although for 1 and 2 wt. % loadings, it becomes difficult to define the maximum temperature. There is no appreciable change in T_g_ values as MWCNT quantity is increased. Similar behaviour has been reported for PET/MWCNT nanocomposites by Santoro et al. [66] and for PE/MWCNT nanocomposites by Logakis et al. [61] who incorporated up to 5 wt. % of MWCNT and did not observe any change in the values of T_g_. 

## 4. Conclusions 

In this study, PET composites were prepared using three different dimensional carbon fillers. These composites were fabricated by a melt compounding method followed by compression moulding and then a quenching process to reduce the crystallization behaviour. The viscoelastic properties of PET were investigated, considering the dimensionality and loading of the carbon fillers. It was observed that 1D nano-filler (MWCNT) was found to affect the E′ at very low loadings (0.1 wt. %) in comparison to 2D nano-filler with (GNP) and 3D micro-filler (graphite) fillers that exhibited similar E′ behaviour at higher loadings (2 wt. %). SEM showed some occurrences of agglomeration, poor distribution, debonding and rolling up (of both MWCNT and GNP) in the PET composites at higher filler loadings, resulting in a reduction in the E′ values. 

Nevertheless, the T_g_ of all the composites remained essentially unaffected by either the dimensionality of the carbon fillers or their loadings. The tan δ value of the PET composites containing 1D nano-filler is ~0.3, which is significantly lower than that of PET composites containing 2D nano-filler (tan δ = 1.02) and 3D micro-filler (tan δ = 1.08), reductions of ≈240% and 260%, respectively; indicative of greater chain segment constraint in the interfacial region between PET and the MWCNT. These results suggest that fillers with lower dimensionality have a more significant effect on the viscoelastic properties of PET composites. However, all composites samples exhibited noteworthy changes in their viscoelastic properties due to both carbon filler adding and to cold crystallization behaviour. 

## Figures and Tables

**Table 1:** Selected DMTA data for E′, T_g_ and tan δ of PET/graphite microcomposites.

Graphite (wt. %)	E′ at 25 °C (MPa)	E′ at 100 °C (MPa)	T_g_ (°C)	tanδ at T_g_
0	1330 ± 92	10.0 ± 2.5	80.5 ± 1.3	1.15 ± 0.1
2	1417 ± 130	10.0 ± 4.0	80.9 ± 0.3	1.08 ± 0.1
5	1536 ± 177	13.0 ± 6.0	80.8 ± 0.6	1.00 ± 0.1
10	1893 ± 119	11.0 ± 3.0	82.4 ± 1.2	1.02 ± 0.2
15	1928 ± 142	13.5 ± 0.8	82.7 ± 1.5	0.88 ± 0.1

**Table 2:** Selected DMTA data for E′, T_g_ and tan δ of PET/GNP nanocomposites.

GNP (wt. %)	E′ at 25 °C (MPa)	E′ at 100 °C (MPa)	T_g_ (°C)	tanδ at T_g_
0	1330 ± 92	10. 0 ± 2.5	80.5 ± 1.3	1.15 ± 0.10
2	1447 ± 88	22.9 ± 2.2	80.0 ± 0.9	1.02 ± 0.30
6	2308 ± 62	44.6 ± 5.2	82.7 ± 0.5	0.93 ± 0.02
8	2095 ± 84	129.4 ± 14	83.5 ± 0.7	0.69 ± 0.04
10	1782 ± 33	120.0 ± 44	82.1 ± 0.9	0.69 ± 0.10

**Table 3:** Selected DMTA data for E′, T_g_ and tan δ of PET/MWCNT nanocomposites.

MWCNT (wt. %)	E′ at 25 °C (MPa)	E′ at 100 °C (MPa)	T_g_ (°C)	tanδ at T_g_
0	1330 ± 92	10.0 ± 2.5	80.5 ± 1.3	1.15 ± 0.1
0.1	1668 ± 111	20.0 ± 1.4	82.0 ± 2.0	0.98 ± 0.2
0.2	1396 ± 108	8.0 ± 1.7	83.0 ± 2.0	1.16 ± 0.1
1	1627 ± 123	46.0 ± 1.3	81.0 ± 1.0	0.55 ± 0.02
2	1550 ± 167	169 ± 43	84.4 ± 0.6	0.30 ± 0.02

## Data Availability

Not applicable.

## Supplementary Materials

The following supporting information can be downloaded at: https://www.mdpi.com/article/10.3390/polym14122440/s1, Figure S1: Fracture surfaces morphology of PET/graphite micro-composites at low and high magnifications with 2 wt. % graphite. Figure S2: Fracture surfaces morphology of PET/GNP nanocomposites at low and high magnifications with 2 wt. % GNP. Figure S3: Fracture surfaces morphology of PET/ MWCNT nanocomposites at low and high magnifications with 1 wt. % MWCNT. Table S1: Comparison of the degree of crystallinity. Figure S4: FTIR spectra of investigated carbon fillers i.e. MWCNT, GNP and Graphite. Figure S5: DSC curves (heating/cooling rate 10 °C/min) for PET; showing the first heating scan (b), the second heating scan. Figure S6: Degree of crystallinity (DoC) of PET/carbon composites at 2 wt. % carbon fillers. Figure S7: Second DMTA curve of dynamic storage modulus (E′) as a function of temperature for the PET matrix specimen shown in Figure 2 (in the manuscript). The insert shows first heating run on DSC for the same (now crystallized) PET specimen. Figure S8: Comparison of E′ data from references with the present experimental results. Figure S9: Comparison of E′ data from references with the present experimental results. Figure S10: Comparison of E′ data from references compared with the present experimental results.
