# Supplementary material for: Influence of Carbon Micro- and Nano-Fillers on the Viscoelastic Properties of Polyethylene Terephthalate
Source: Polymers (Basel). 2022 Jun 16;14(12):2440. doi: 10.3390/polym14122440 (PMC9227514; doi:10.3390/polym14122440)
Supplement: Supplementary file 1 [file polymers-14-02440-s001.zip › polymers-1773428-supplementary.pdf]

Supporting Information

Influence of Various Carbon Fillers Dimensionality on Viscoelastic Properties of Polyethylene Terephthalate Matrix

Basheer A. Alshammari[1*], Arthur N. Wilkinson[2], Bandar M. AlOtaibi[3] and Mohammed F. Alotibi[1]

[1]Material Research Institute, King Abdulaziz City for Science and Technology, P.O. Box 6086, Riyadh 11442, Saudi Arabia

[2]Materials Science Centre and North West Composites Centre, School of Materials, The University of Manchester, Grosvenor Street, Manchester M13 9PL, UK

[3]The National Center for Energy Storage Technologies, King Abdulaziz City for Science and Technology, P.O. Box 6086, Riyadh 11442, Saudi Arabia

* Correspondence should be addressed to Basheer A. Alshammari; bshammari@kacst.edu.sa

S1. Morphological and Crystallinity Characterizations

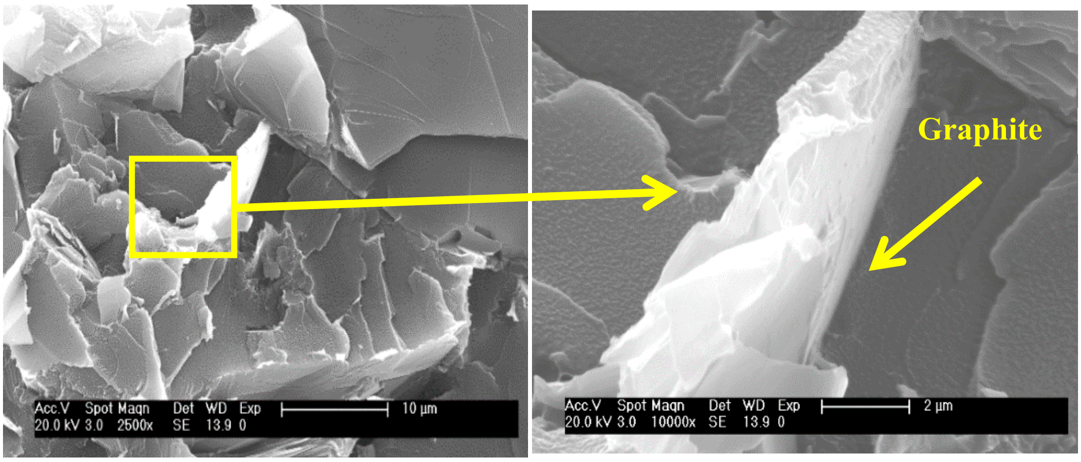

Figure S1. Fracture surfaces morphology of PET/graphite micro-composites at low and high magnifications with 2 wt. % graphite.

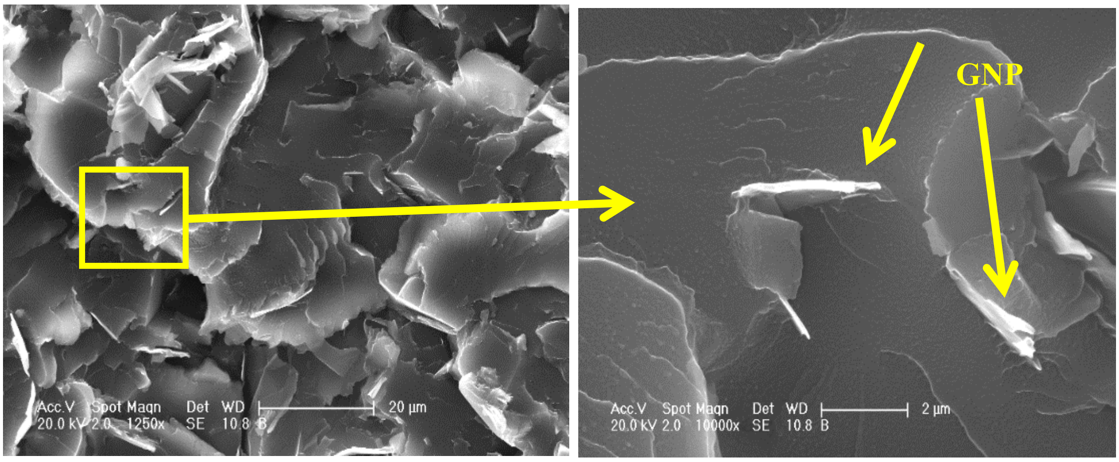

Figure S2. Fracture surfaces morphology of PET/GNP nanocomposites at low and high magnifications with 2 wt. % GNP.

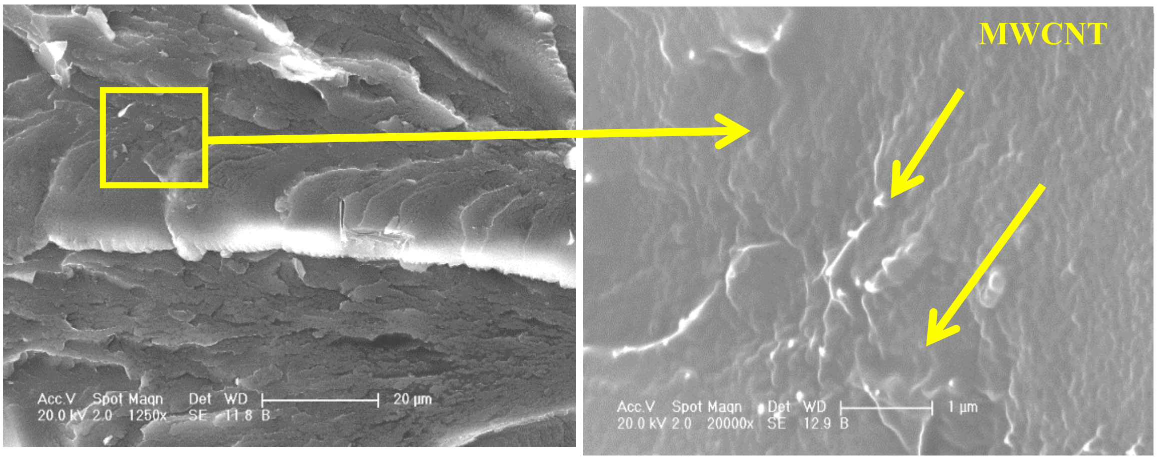

Figure S3. Fracture surfaces morphology of PET/ MWCNT nanocomposites at low and high magnifications with 1 wt. % MWCNT.

Table S1. Comparison of the degree of crystallinity

**Samples**	**Degree of crystallinity (%)**
Pure PET	11.8 ±0.8
**2 wt.% Graphite**	15.6 ±1.6
**2 wt.% GNP**	19.3 ±0.3
**2 wt.% MWCNT**	22.5 ±1.8

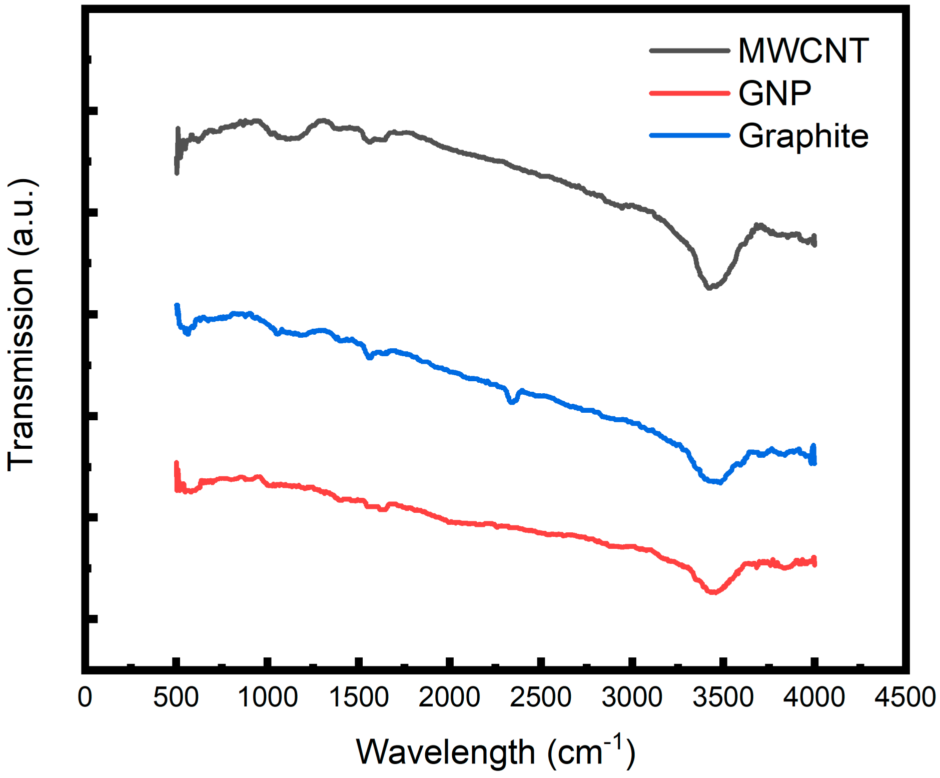

Figure S4. FTIR spectra of investigated carbon fillers i.e. MWCNT, GNP and Graphite.

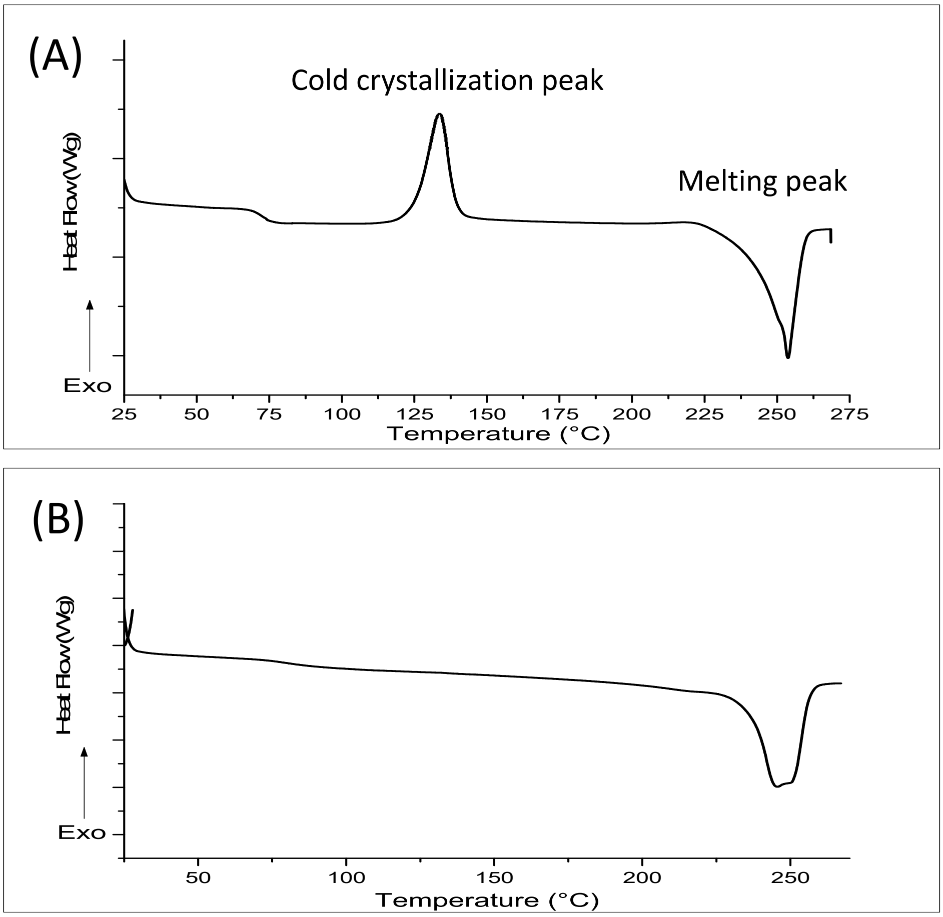

Figure S5. DSC curves (heating/cooling rate 10 °C/min) for PET; (A) showing the first heating scan, (B) the second heating scan.

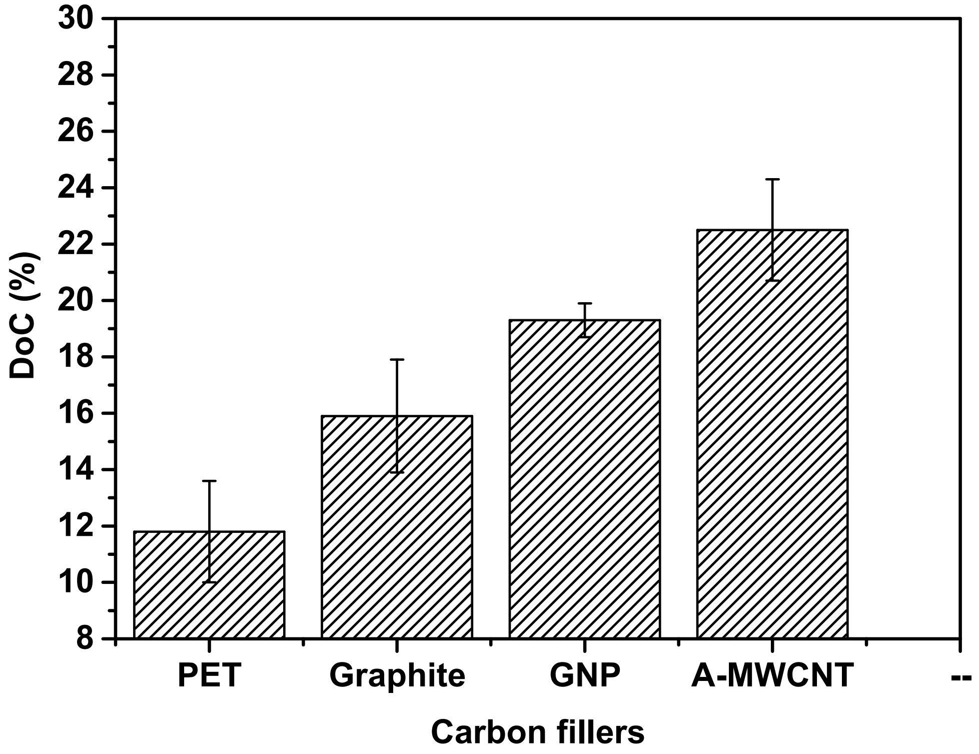

Figure S6. Degree of crystallinity (DoC) of PET/carbon composites at 2 wt. % carbon fillers.

S2. Dynamical Mechanical Properties

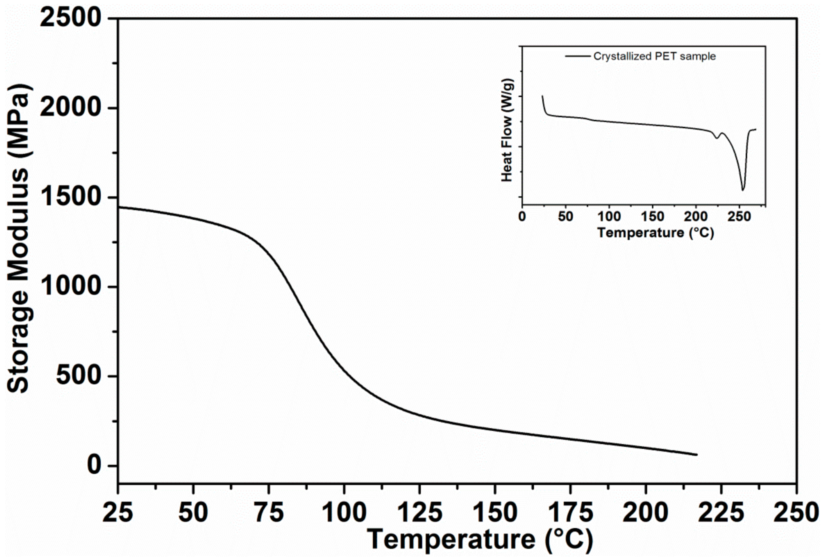

Figure S7. Second DMTA curve of dynamic storage modulus (E') as a function of temperature for the PET matrix specimen shown in Figure 2 (in the manuscript). The insert shows first heating run on DSC for the same (now crystallized) PET specimen.

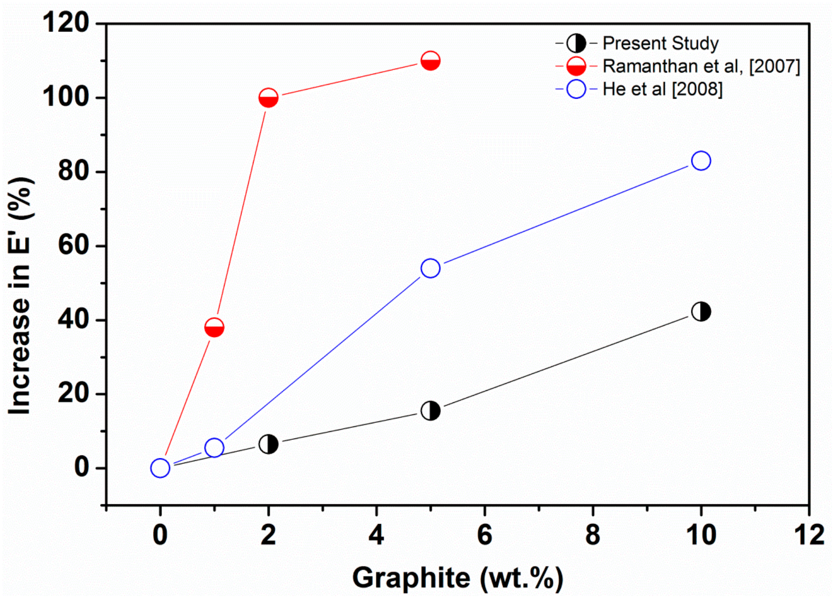

Figure S8. Comparison of E′ data from reference [1] (PMMA matrix) and [2] (PVDF matrix) with the present experimental results.

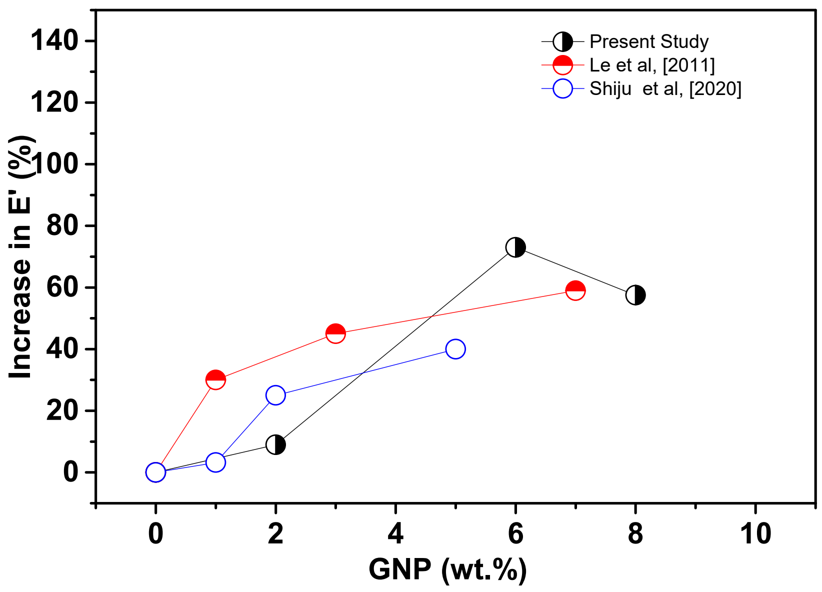

Figure S9. Comparison of E′ data from reference [3] (PMMA matrix) and [4] (PET matrix) with the present experimental results.

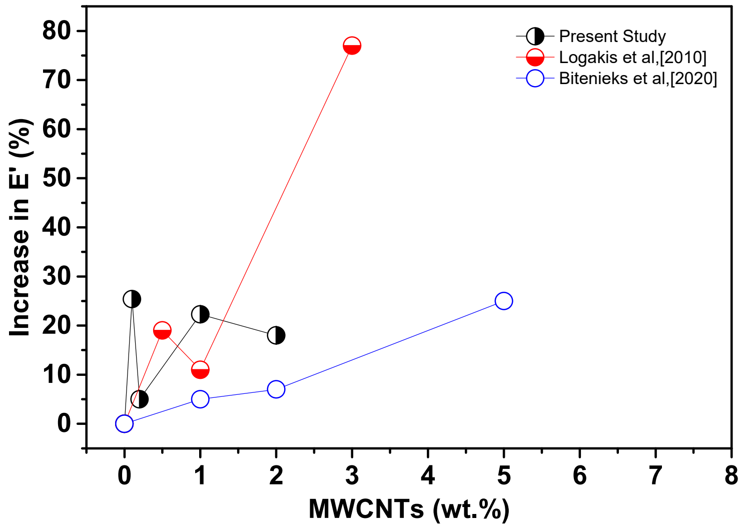

Figure S10. Comparison of E′ data from references [5] (PP matrix) and [6] (PE matrix) compared with the present experimental results.

References:

[1] T. Ramanathan, S. Stankovich, D. Dikin, H. Liu, H. Shen, S. Nguyen, and L. Brinson, "Graphitic nanofillers in PMMA nanocomposites—an investigation of particle size and dispersion and their influence on nanocomposite properties," Journal of Polymer Science Part B: Polymer Physics, vol. 45, pp. 2097-2112, 2007.

[2] F. He, J. Fan, and S. Lau, "Thermal, mechanical, and dielectric properties of graphite reinforced poly (vinylidene fluoride) composites," Polymer Testing, vol. 27, pp. 964-970, 2008.

[3] Shiju, J., Al-Sagheer, F., & Ahmad, Z. (2020). Thermal mechanical properties of graphene nano-composites with Kevlar-Nomex copolymer: A comparison of the physical and chemical interactions. Polymers, 12(11), 2740.

[4] Li, Meilu, Jeong, and Y. Gyu, "Poly (ethylene terephthalate)/exfoliated graphite nanocomposites with improved thermal stability, mechanical and electrical properties," Composites Part A: Applied Science and Manufacturing, vol. 42, pp. 560-566, 2011.

[5] Bitenieks, J., Merijs Meri, R., Zicans, J., & Buks, K. (2020). Dynamic Mechanical, Dielectrical, and Rheological Analysis of Polyethylene Terephthalate/Carbon Nanotube Nanocomposites Prepared by Melt Processing. International Journal of Polymer Science, 2020.

[6] E. Logakis, E. Pollatos, C. Pandis, V. Peoglos, I. Zuburtikudis, C. Delides, A. Vatalis, M. Gjoka, E. Syskakis, and K. Viras, "Structure–property relationships in isotactic polypropylene/multi-walled carbon nanotubes nanocomposites," Composites Science and Technology, vol. 70, pp. 328-335, 2010.
